# Effects of *PPP1R1B* (*DARPP-32*) Polymorphism on Feedback-Related Brain Potentials Across the Life Span

**DOI:** 10.3389/fpsyg.2013.00089

**Published:** 2013-03-04

**Authors:** Dorothea Hämmerer, Gudio Biele, Viktor Müller, Holger Thiele, Peter Nürnberg, Hauke R. Heekeren, Shu-Chen Li

**Affiliations:** ^1^Center for Lifespan Psychology, Max Planck Institute for Human DevelopmentBerlin, Germany; ^2^Department of Psychology, Technische Universität DresdenDresden, Germany; ^3^Department of Education and Psychology, Freie Universität BerlinBerlin, Germany; ^4^Institute of Psychology, University of OsloOslo, Norway; ^5^Cologne Center for Genomics, University of CologneCologne, Germany; ^6^Center for Molecular Medicine Cologne, University of CologneCologne, Germany; ^7^Cologne Excellence Cluster on Cellular Stress Responses in Aging-Associated Diseases, University of CologneCologne, Germany

**Keywords:** *PPP1R1B* (DARPP-32), dopamine, reward, reinforcement learning, child development, aging

## Abstract

Maximizing gains during probabilistic reinforcement learning requires the updating of choice – outcome expectations at the time when the feedback about a specific choice or action is given. Extant theories and evidence suggest that dopaminergic modulation plays a crucial role in reinforcement learning and the updating of choice – outcome expectations. Furthermore, recently a positive component of the event-related potential about 200 ms (P2) after feedback has been suggested to reflect such updating. The efficacy of dopaminergic modulation changes across the life span. However, to date investigations of age-related differences in feedback-related P2 during reinforcement learning are still scarce. The present study thus aims to investigate whether individual differences in the feedback-related P2 would be associated with polymorphic variations in a dopamine relevant gene *PPP1R1B* (also known as *DARPP-32*) and whether the genetic effect may differ between age groups. We observed larger P2 amplitudes in individuals carrying the genotype associated with higher dopamine receptor efficacy, i.e., a allele homozygotes of a single nucleotide polymorphism (rs907094) of the *PPP1R1B* gene. Moreover, this effect was more pronounced in children and older adults in comparison to adolescents and younger adults. Together, our findings indicate that polymorphic variations in a dopamine relevant gene are associated with individual differences in brain-evoked potentials of outcome updating and hint at the possibility that genotype effects on neurocognitive phenotypes may vary as a function of brain maturation and aging.

## Introduction

Flexible control of behavior requires the representation, maintenance, and updating of goal-relevant context information. For instance, in real-life situations of choosing between stocks, even though fluctuations in the markets are highly unpredictable, the profit histories, and risk profiles of different stocks are common context information that are considered when making choices. In laboratories, maximizing gains during probabilistic reinforcement learning requires the updating of choice–outcome expectations at the time when feedback about a specific choice is given. Studies using electroencephalography (EEG) have identified event-related potentials that are related to reinforcement learning (see Nieuwenhuis et al., 2004 for a review). Of specific interest here is a positive frontal-centrally distributed component about 200 ms after the feedback for an action or a choice, called feedback-related P2; the amplitude of this component can be modulated by expectancy. For instance, the amplitude of the P2 was found to be larger for unexpected than for expected (cued) stimuli (JieMin et al., 2010). In a related vein, the P2 amplitude seems to reflect stimulus relevancy for context updating in Go-Nogo (Lenartowicz et al., 2010) and task-switching paradigms (Astle et al., 2008). Source estimations implicate the right dorsolateral prefrontal cortex (DLPFC) in the generation of the P2 during context updating (Lenartowicz et al., 2010). Taken together, extant evidence suggests that the feedback-related P2 may reflect flexible updating in changing environments.

Very recently, there is also evidence from a functional brain imaging study, which suggests an involvement of the subcortical dopaminergic system in modulating prefrontally based updating processes. Specifically, D’Ardenne et al. (2012) observed an increased response in the ventral tegmental area (VTA) as well as in the DLPFC during context updating when the participants performed a Go-Nogo task. Moreover, applying TMS over the right DLPFC increased reaction times especially during conditions requiring context updating, confirming the relevance of the right DLPFC for updating (D’Ardenne et al., 2012). Together, these findings indicate that subcortical dopaminergic structures may contribute to DLPFC’s context updating function via a frontal-striatal pathway.

### Lifespan differences in dopaminergic modulation

The dopaminergic systems undergo maturation and senescence across the lifespan. Dopamine modulation in the prefrontal cortex (PFC) as well as subcortical and midbrain regions is less effective during childhood and old age in comparison to other life periods. For instance, there is ample evidence for aging-related declines of pre- and postsynaptic markers of the dopamine system (see Bäckman et al., 2006; Li et al., 2010; Li, 2012 for reviews). Based on cross-sectional estimates, the aging-related decline progresses with a rate of about 10% per decade starting in the third decade of life and affects midbrain (Bannon and Whitty, 1997; Reeves et al., 2002), subcortical (Seeman et al., 1987; Rinne et al., 1990; see also Severson et al., 1982) as well as frontal, cingulate, temporal, parietal, and occipital cortical areas (Kaasinen et al., 2000; Inoue et al., 2001). With respect to child development, the evidence is much scarcer due to obvious reservations of applying invasive methods, such as PET receptor imaging, in these age groups. However, increasing dopamine and dopamine transporter levels in the dorsal striatum have been reported until the age of 9, by which point adult levels are reached (Haycock et al., 2003, see also Seeman et al., 1987). In contrast to the relatively early maturation of the subcortical dopaminergic system, D1 receptor function in the lateral PFC develops much more gradually and only reaches maturation around adulthood (Rothmond et al., 2012). Given lifespan age differences in brain development in general and in dopamine functions in specific, lifespan age differences in evoked brain potentials relevant for reinforcement learning and outcome updating may be expected. Whereas lifespan age differences in the feedback-related negativity have been observed in a few studies (see Eppinger et al., 2011; Hämmerer and Eppinger, 2012 for reviews), developmental and aging-related effects on the feedback-related P2 component have not been investigated systematically.

### Study aims

Taking a candidate gene approach, the aim of this study is thus twofold: (i) to investigate whether polymorphic variations in a dopamine relevant gene may contribute to individual differences in the feedback-related P2 amplitude; (ii) to investigate whether dopamine relevant genotype effects on the feedback-related P2 may interact with age, given that the efficacy of dopamine modulation changes across the lifespan.

To address the first question, we focus on a well-studied molecular candidate for dopamine signaling, the DARPP-32 protein (now also known as *PPP1R1B*, protein phosphatase 1, regulatory inhibitor subunit 1B), which is richly expressed in the striatum. The DARPP-32 protein is phosphorylated by dopamine D1 receptor stimulation, and dephosphorylated by D2 receptor stimulation (Nishi et al., 1997). The protein DARPP-32 is encoded by the *PPP1R1B* gene. The single nucleotide polymorphism (rs907094) in the *PPP1R1B* gene affects the mRNA expression of the protein. A homozygosity of the *PPP1R1B* gene is associated with higher mRNA expression and higher dopamine receptor efficacy. Furthermore, individuals carrying haplotypes of the DARPP-32 gene associated with greater mRNA expression show greater changes in BOLD signal in the striatum, greater frontal-striatal connectivity during cognitive performance, as well as better cognitive performance on executive functions (Meyer-Lindenberg et al., 2007). We thus hypothesized that the amplitude of the feedback-related P2 would be larger in the A homozygotes of the *PPP1R1B* gene than in any G carriers. To address the second question, we compare this effect in four age groups, sampled across the lifespan. We expected the potential effect of the *PPP1R1B* polymorphism on the P2 amplitude to be larger in age groups with reduced dopaminergic modulation, that is, in children and older adults. Such a result would be in line with previous findings of larger effects of individual differences in genetic variations in populations with suboptimal neuromodulation (e.g., Nagel et al., 2008; Li et al., 2013).

## Materials and Methods

### Sample

Only male subjects were included in the present analyses given that endogenous fluctuations in estradiol during the menstrual cycle alter dopamine synthesis (Jacobs and D’Esposito, 2011). Our sample included 20 children (9–11 years), 23 teenagers (13–14 years), 22 younger adults (20–30 years), and 21 older adults (65–75) recruited from the participant pools of the Center for Lifespan Psychology, Max Planck Institute for Human Development (for a previous report on parts of this dataset, see Hämmerer et al., 2011). All participants were residents of Berlin, Germany. None reported a history of medical, neurological, psychiatric disease, or head injury. All participants or parents of the participants gave informed consent, and the study was approved by the local ethics board. Participants were paid for their participation in the study (10€ for the first, and 7€ for every following hour of the experiment).

### Genotyping

Saliva was collected from the participants using the Oragene^®^ DNA sample collection kit (ON, Canada). DNA was extracted from saliva using standard techniques. TaqMan probes for the single nucleotide polymorphism (SNP) genotyping were designed and synthesized by Applied BioSystems (Foster City, CA, USA). The SNP rs907904 for the *PPP1R1B* gene was selected based on previous studies (cf. Frank et al., 2007b). The breakdowns of the *PPP1R1B* genotypes were 39:43:4 (AA/AG/GG) in our sample and were in the Hardy–Weinberg equilibrium, χ^2^(1) = 3.39, *p* > 0.05. Given that the frequency of the reference allele (G) is low, similar to previous reports (Frank et al., 2007b), we examined genotype effects by comparing A homozygotes (AA; *n* = 39) to AG and GG carriers combined (any G; *n* = 47).

### Experimental procedure

During EEG recordings, participants were comfortably seated in an electrically and acoustically shielded room. The distance to the computer screen was 80 cm. In a reinforcement learning task, participants were presented with pairs of Japanese characters that were each associated with probabilistic gains and losses. Feedback was presented 500 ms after the choice. However, within each pair, one symbol had a higher probability of leading to a gain than the other symbol (cf. Frank et al., 2007b). Subjects were asked to maximize gains by identifying the option with a greater gain probability in each pair. There were three types of pairs, differing with respect to the distinctiveness of the gain probabilities between the two symbols (85 vs. 15%, 75 vs. 25%, 65 vs. 35%). After each block of 60 trials, the percentage of choices in which the better option within each pair had been chosen in that block was assessed. Learning criteria for each block were set to choosing the better symbol at least in 65, 70, and 75% of the pair presentations for the least, medium, and most distinct pairs, respectively. When participants had reached the learning criteria for all three pair types, a new set of three pairs was introduced. A maximum of three sets of three pair types could be learned in this task. This resulted in different numbers of blocks across the participants. The minimum number of blocks was three blocks. The task stopped after a maximum of 12 blocks independent of whether all three sets of three pairs had been completed. This approach was chosen to ensure that despite the expected age differences in the speed of learning, behavioral, and electrophysiological data collected during the task reflected the learning from negative and positive feedbacks in all age groups (for further details on the task procedure, see Hämmerer et al., 2011).

### EEG recordings and data preparation

Electroencephalography was recorded from 64 Ag/AgCl electrodes placed according to the 10–10 system in an elastic cap (Braincap, BrainVision), using BrainVision Recorder. The sampling rate was 1000 Hz with a bandpass filter applied in the range of 0.01–250 Hz. EEG recordings were referenced online to the right mastoid. The ground was positioned above the forehead. Impedances were kept below 5 kΩ. Vertical and horizontal electrooculograms were recorded next to each eye and below the left eye.

**Figure 1:**
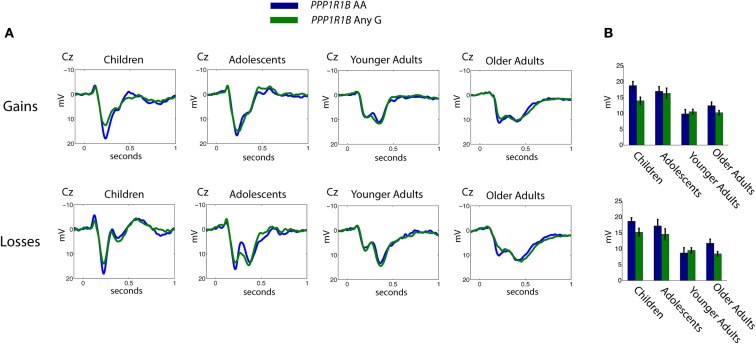
**(A)** Grand average of the stimulus-locked ERPs to positive and negative feedback for the AA (higher striatal dopamine receptor efficacy) and “any G” genotypes (lower striatal dopamine receptor efficacy) for rs907094 across the four age groups. **(B)** Inserts indicate mean amplitude for each age group of the individually defined P2 peak at electrode Cz. Error bars denote 1 SE of the mean.

Using BrainVision Analyzer, the recorded data were referenced to an average reference. Using the Fieldtrip software package (for more details, see http://www.ru.nl/fcdonders/fieldtrip), the data were then segmented into epochs of 2 s before and 2.5 s after the onset of the feedback. Epochs or channels with severe muscular artifacts or saturated recordings were excluded manually. The prepared data were subjected to an ICA decomposition using EEGLAB (Delorme and Makeig, 2004) for artifact rejection. ICA components of ocular and muscular artifacts were removed from the data. The recombined data were bandpass-filtered in the range of 0.5–25 Hz and epoched 1000 ms after and 100 ms before the onset of the feedback symbols. Baseline corrections were applied on the epoched data with respect to the 100 ms pre-stimulus baseline. ERPs were obtained by averaging across all artifact-free trials for each electrode and condition for each participant. Amplitudes of the P2 following the feedback symbols were defined as the most positive peaks in the individual averages in the time windows 100–250 ms (Nieuwenhuis et al., 2002; see Falkenstein et al., 2002; Johnstone et al., 2007; Eppinger et al., 2008, 2009; for comparable time windows in developmental studies of EEG components related to performance monitoring). We focused on peak instead of mean area measures of a specified time window since a comparison of mean measures across different age groups might be biased by age differences in the slope of the ERP (e.g., Jonkman et al., 2003, see also Figure 1). Also, adult age differences in the slope of ERPs have been shown to be independent of age differences in temporal jitter of the single ERPs, which could have been considered to reflect in slope differences in the averaged ERPs (Walhovd et al., 2008). P2 Amplitude was measured at frontocentral electrodes (i.e., average across FC3, FCz, FC4, C3, Cz, C4 electrodes). These localizations are in line with the scalp distributions observed in prior developmental studies (e.g., Jonkman, 2006; Müller et al., 2008).

### Data analysis

The data were analyzed using SPSS 15.0 (SPSS Inc., Chicago, IL, USA) and SAS 9.1 for Windows (SAS Institute, Cary, NC, USA). Given that age groups differ in the amount of intragroup between-person variance, comparisons across age groups were done using the PROC MIXED procedure, which is robust to variance heterogeneity between age groups. The intraclass correlation coefficient (ICC) was calculated as the effect size indicator for ANOVA. Cohen’s *d* was calculated as the effect size indicator for planned contrasts and pairwise comparisons across the age groups.

## Results

**Figure 2:**
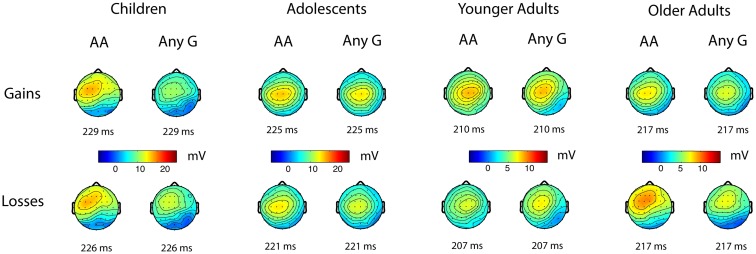
**Scalp topographies of the stimulus-locked ERPs to positive and negative feedback for the AA (higher striatal dopamine receptor efficacy) and “any G” (lower striatal dopamine receptor efficacy) genotypes for rs907094 across the four age groups**. Timings below the maps are given relative to stimulus onset. Maps are based on mean amplitudes of a 50 ms interval around indicated time. Please note the different scale for children and adolescents.

Figure 1 displays the grand averages of stimulus-locked ERPs to gain and loss feedback split up for the *PPP1R1B* “any G” and AA genotypes, whereas Figure 2 shows the corresponding scalp distributions of the stimulus-locked ERPs in the P2 time window. As shown in Figure 2, the P2 peak (see Materials and Methods) was largest in all age groups at the frontocentral electrodes. Given that the A homozygotes are associated with higher striatal dopamine receptor efficiency, we expected P2 amplitudes to be larger for the AA carriers than for any G carriers.

Indeed, in a three-way ANOVA with the factor gain-loss as within-subject factor, and age and genotype as between-subject factors, we observed a reliable main effect of *PPP1R1B*, *F*(1,27.1) = 4.72, *p* < 0.05, ICC = 0.39. As evident in Figure 1, the main effect of age was also significant, *F*(3,16.6) = 13.20, *p* < 0.05, ICC = 0.84, reflecting larger P2 amplitudes in children and adolescents (group contrast: children – adolescents – younger adults – older adults 1 1 −1 −1; *t* = 6.00, *p* < 0.05, *r* = 0.76). In addition, P2 amplitudes were reliably higher in the gain as compared to the loss condition, *F*(1,21.7) = 5.25, *p* < 0.05, ICC = 0.44. The overall two-way age × *PPP1R1B* interaction effect was not reliable, *F*(3,16.6) = 1.23, *p* = 0.33, likely due to limited power.

As a next step, we applied planned contrasts to test our second hypothesis of an amplification of the genetic effect in age groups with reduced dopamine levels. Specifically, a contrast testing for a U-shaped pattern of the *PPP1R1B* effect across the lifespan was performed (cf. Ryan, 1959). The planned contrast yielded an effect of greater *PPP1R1B* influences on P2 amplitude in children and older adults [group contrasts: age (children – adolescents – younger adults – older adults) × *PPP1R1B* (AA – any G) 1 −1 −1 1 −1 1 1 −1; *t* = 1.65, *p* = 0.05, *d* = 0.37). As can also be seen in Figure 1, *t*-tests comparing the *PPP1R1B* subgroups within age groups confirmed the specificity of this effect for children and older adults: (children: gain P2 *t* = 3.2, *p* < 0.05, loss P2 *t* = 2.1, *p* = 0.05; adolescents: gain P2 *t* = 0.52, *p* = 0.61, loss P2 *t* = 1.2, *p* = 0.26; younger adults: gain P2 *t* = 0.25, *p* = 0.82, loss P2 *t* = 0.22, *p* = 0.84; older adults: gain P2 *t* = 1.6, *p* = 0.15, loss P2 *t* = 2.1, *p* < 0.05).

Similar to prior studies (Frank et al., 2007a; Lenartowicz et al., 2010), we did not observe a relationship between the average P2 amplitude after losses and gains and the behavioral performance (mean accuracy, defined as the proportion of choices of the better option irrespective of gain or loss outcomes) in the two genotype groups (regression mean accuracy on P2 amplitude: AA carrier after negative feedback: *beta* = −0.04, ns; Any G carrier after negative feedback: *beta* = −0.03, ns; AA carrier after positive feedback: *beta* = 0.01, ns; Any G carrier after positive feedback: *beta* = 0.19, ns). This might be due to the fact that the updating processes in the P2 only reflect flexibility with respect to new information and is neutral with respect to the appropriate use of the new information. However, a relation to behavioral parameters might be expected when a change in the flexibility is behaviorally adaptive. In the present probabilistic reinforcement learning task, it is, for instance, adaptive to reduce the updating based on loss feedback in later stages of learning. Once the better option is identified, the infrequent, improbable losses (in this task about 25%) on this option should not be considered much during updating. In line with this reasoning, we observed a positive relationship of the decrease in P2 amplitude to losses after reaching the learning criterion (about 75% correct choices) with the mean accuracy (defined as the proportion of choices of the better option, irrespective of gain and loss outcomes). Subjects with higher mean accuracy exhibited a reduced updating response to negative feedback after reaching the learning criterion. Of particular interest, this relationship was stronger in subjects with the AA genotype, suggesting that higher dopamine receptor efficiency supports the flexibility in the updating process (regression mean accuracy on difference P2 amplitude during and after learning, controlling for age differences in P2 amplitude: AA carrier after negative feedback: *beta* = 0.47, *p* < 0.01; Any G carrier after negative feedback: *beta* = 0.13, ns; AA carrier after positive feedback: *beta* = −0.01, ns; Any G carrier after positive feedback: *beta* = 0.19, ns.).

## Discussion

The present study provides evidence for genotype effects on the P2 amplitude during outcome updating. Homozygotes of the A allele of the *PPP1R1B* gene (SNP rs907094), who presumably are associated with higher frontostriatal function, show larger P2 responses to feedbacks during reinforcement learning. Furthermore, this observed genotype effect is stronger in individuals with lower efficacy of dopamine signaling, i.e., in children and older adults.

### Genetic variations in dopaminergic modulation and outcome updating

Theories of cognitive control in general (e.g., Braver and Cohen, 2000; Braver et al., 2001; Miller and Cohen, 2001; O’Reilly and Frank, 2006), of reinforcement learning (e.g., Holroyd and Coles, 2002; Daw et al., 2005; Schultz, 2007, 2010), or of value-based decision making (e.g., Frank and Claus, 2006; Rangel et al., 2008; Frank et al., 2009) converge on the role of the subcortical dopamine system in providing a learning signal for updating goal-relevant information. However, as of now, there is only indirect evidence of a link between (subcortical) dopamine release and prefrontally generated feedback-related ERP components. For instance, a gene coding for Catechol-*O*-methyltransferase (COMT), an enzyme that degrades dopamine in the PFC has been found to affect the amplitude of the error-related positivity, which is assumed to reflect the expectedness of an outcome (Campbell et al., 1979). Met homozygotes of the *COMT* gene polymorphism (rs4680), with their presumably higher dopamine levels in the PFC, showed a larger error-related positivity than the Val carriers (Frank et al., 2007a; Heitland et al., 2012). Moreover, a larger FRN, a component indicating the evaluation of outcomes in light of task goals (Holroyd et al., 2006), is observed in carriers of the DAT1 gene 9-repeat allele with presumably higher dopamine levels in the striatum (Heitland et al., 2012). A more recent pharmacological study tested more directly whether dopamine modulates feedback-related electrophysiological responses. Santesso et al. (2009) showed that a dose of pramipexole resulted in higher FRN amplitudes after positive feedback, which is suggested to imply a reduced FRN sensitivity to positive prediction errors when the phasic dopamine response is reduced. Finally, Carlson et al. (2011) found that individuals who showed greater BOLD activations in the mesocorticolimbic reward circuit, including the ventral striatum and the medial PFC, also showed higher amplitudes in a measure of the FRN to negative feedback. Although this study has not directly tested the relation between the FRN and dopamine levels or receptor density, it shows that individuals with larger FRN to negative feedback also showed greater striatal BOLD activations.

To summarize, current evidence shows that feedback-related ERP components vary with genetic polymorphisms relevant for dopaminergic modulation, that dopaminergic drugs can alter feedback-related ERPs, and that activation in subcortical structures is related to ERP amplitudes. Together these findings might be a starting point to piece together information on how dopaminergic modulation might relate to functional processes reflected in ERP components. We add to these findings by showing that the P2 amplitude during outcome updating varies with polymorphisms of the *PPP1R1B* gene.

### Age differences in genotype effects on evoked brain potentials of outcome updating

We observed a larger effect of *PPP1R1B* genotype on feedback-related P2 in children and older adults, whose dopamine levels are lower, either because the dopamine systems are still maturing or because they have started to decline. This result is of interest and suggests that changes in brain resources at the anatomical or neurochemical levels during maturation or senescence may modulate genotype-phenotype relations in different life periods, since brain mechanisms are the “intermediate phenotypes” (Meyer-Lindenberg and Weinberger, 2006) between genetic expressions in the central nervous system and behavioral phenotypes (Lindenberger et al., 2008; Li et al., 2013). Genes related to the neurotransmitter dopamine represent a case in point. Evidence from clinical (Mattay et al., 2003) and animal (Vijayraghavan et al., 2007) studies as well as neurocomputational simulations (Li and Sikström, 2002) suggest that the relation between dopamine levels and cognitive performance follows an inverted-U function (see Cools and D’Esposito, 2011, for review). The non-linear function relating dopamine modulation to cognitive performance predicts that genetic effects on cognition would be more apparent when dopamine levels recede from an optimal level, such as in childhood or old age or in situations when the natural dopamine level is perturbed by excessive stress or stimulants that affect neuromodulation (Lindenberger et al., 2008; Li et al., 2010). So far, findings from less than a handful of studies on aging lend preliminary support to this resource-modulation hypothesis. For instance, older adults’ spatial working memory and executive functioning were associated with individual differences in genetic predispositions of the *COMT* gene, which affects dopamine levels in the PFC, whereas the genetic effect in younger adults was limited (Nagel et al., 2008; Störmer et al., 2012). Despite this accumulating evidence, it should be kept in mind that age by genotype interactions provide only an indirect hint at lifespan differences in the dopaminergic system, including long-term adaptations to changes in receptor density and interactions with other transmitters, that may influence the effects of dopamine relevant genes on the behavioral and intermediate brain phenotypes in different age groups. As with any genetic association study, the observed relation between genotype and intermediate brain phenotype need to be verified in future independent samples for the association to be considered as established.

### Individual differences in behavioral measures of outcome updating

In the present task, it is adaptive to reduce the updating based on loss feedback in later stages of learning, as the infrequent improbable losses on the better option at later stages of learning should not guide behavior. In line with this reasoning, we observed that higher performance (i.e., percent correct choices) was associated with a higher decrease of P2 amplitude after learning for losses, but not for gains. Moreover, this effect was stronger for subjects with the AA genotype, which again hints at the role of dopamine signaling in a flexible updating process. This finding, however, needs to be considered in light of other, related studies which did not observe a relationship between P2 amplitude and accuracy (Frank et al., 2007a; Lenartowicz et al., 2010). One possible reason for the lack of behavioral correlates with P2 amplitude might be that the updating rather represents flexibility with respect to new information and is neutral with respect to the appropriate use of the new information. However, if such flexibility is behaviorally adaptive, as it is in the case in ignoring rare losses, a relation to behavioral parameters should be expected.

## Conclusion

Our findings of a dopamine related genotype effect (*PPP1R1B*) on the P2 amplitude hint at a link between dopamine modulation and outcome updating during reinforcement learning. Individuals carrying the genotype associated with higher striatal dopamine receptor efficacy and fronto-striatal connectivity showed higher P2 responses to feedback during reinforcement learning. Furthermore, this observed genotype effect is stronger in individuals with lower subcortical and/or frontal dopamine levels, i.e., in children and older adults. We hope that these results encourage further research on the link between frontostriatal dopaminergic modulation and functional differences in ERPs and behavior during reinforcement learning. At the same time this suggests a need to consider lifespan age differences in brain functions when investigating genotype-phenotype relations.

Finally, it should also be underscored that, despite current and accumulating evidence suggesting a role of dopamine in affecting feedback-related ERPs, it is conceivable that the observed links (in the current and other studies) between dopamine genotype effect and ERP components of reinforcement learning are less direct than often assumed and may be mediated by interactions between transmitter systems. For instance, it has been suggested that a co-release of glutamate from midbrain dopaminergic neurons might underlie midbrain-prefrontal interactions in the sub-second range as the effects of dopamine release in the PFC unfold too slowly (in the time range of seconds to minutes) to provide an effective updating of prediction errors (see Lapish et al., 2007 for a review, Lavin et al., 2005). Also, other than the frontal-striatal pathways, the striatum also projects to the nucleus basalis in the forebrain, which projects via cholinergic fibers to the cortex and might hence provide another route from the ventral striatum to the PFC (for a review, see Haber and Knutson, 2010). Testing these routes that link subcortical dopamine and prefrontal ERP generators, however, is not within the purview of the current study and needs to be followed-up in future research.

## Conflict of Interest Statement

The authors declare that the research was conducted in the absence of any commercial or financial relationships that could be construed as a potential conflict of interest.
